# Supplementary material for: CXCL9, CXCL10, and CXCL11; biomarkers of pulmonary inflammation associated with autoimmunity in patients with collagen vascular diseases–associated interstitial lung disease and interstitial pneumonia with autoimmune features
Source: PLoS One. 2020 Nov 2;15(11):e0241719. doi: 10.1371/journal.pone.0241719 (PMC7605704; doi:10.1371/journal.pone.0241719)
Supplement: S1 Table — CK: Creatine kinase; SSc: systemic sclerosis; RA: rheumatoid arthritis; PM: polymyositis; MPA: microscopic polyangiitis; MCTD: mixed connective tissue disease; SjS: Sjögren's syndrome. (DOCX) [file pone.0241719.s001.docx]

S1 Table. Organ involvement in CVD–ILD

|  | CVD | Severity of organ involvement | Extrathoracic organ involvement |
| --- | --- | --- | --- |
| 1 | SSc | Lung > other organs | Finger skin thickening |
| 2 | SSc | Lung > other organs | Finger skin thickening, Raynaud's phenomenon |
| 3 | SSc | Lung > other organs | Finger skin thickening |
| 4 | SSc | Lung > other organs | Finger skin thickening |
| 5 | SSc | Lung > other organs | Finger swelling and skin thickening |
| 6 | RA | Lung > other organs | Joint pains in fingers |
| 7 | RA | Lung > other organs | Joint pains in fingers, wrists, and elbows |
| 8 | RA | Lung > other organs | Joint pains in fingers |
| 9 | RA | Lung > other organs | Joint pains and swelling in fingers, wrists, shoulder |
| 10 | PM | Lung > other organs | Skin lesions, CK levels elevation |
| 11 | PM | Lung > other organs | Muscle weakness, CK levels elevation |
| 12 | MPA | Lung | None |
| 13 | MPA | Lung | None |
| 14 | MCTD | Lung > other organs | Edema of hands, Synovitis, Raynaud's phenomenon, Acrosclerosis |
| 15 | MCTD | Lung > other organs | Synovitis, Raynaud's phenomenon, Acrosclerosis |
| 16 | SjS | Lung > other organs | Dry eyes |

CK: Creatine kinase; SSc: systemic sclerosis; RA: rheumatoid arthritis; PM: polymyositis; MPA: microscopic polyangiitis; MCTD: mixed connective tissue disease; SjS: Sjögren's syndrome.
