# Supplementary material for: CXCL9, CXCL10, and CXCL11; biomarkers of pulmonary inflammation associated with autoimmunity in patients with collagen vascular diseases–associated interstitial lung disease and interstitial pneumonia with autoimmune features
Source: PLoS One. 2020 Nov 2;15(11):e0241719. doi: 10.1371/journal.pone.0241719 (PMC7605704; doi:10.1371/journal.pone.0241719)
Supplement: S2 Table — CCL: CC chemokine ligand; CXCL: C-X-C motif chemokine; Fas-L: fas-ligand; IL: inter-leukin; TNF: tumor necrosis factor; TNFSF14: tumor ne-crosis factor superfamily member. *p < 0.05. (DOCX) [file pone.0241719.s002.docx]

S2 Table. Associations between serum and BALF biomarker levels.

|  | rs | p-value |
| --- | --- | --- |
| CCL3 | 0.20 | 0.074 |
| CCL7 | −0.16 | 0.151 |
| CCL17 | 0.39 | <0.001* |
| CXCL9 | 0.43 | <0.001* |
| CXCL10 | 0.39 | <0.001* |
| CXCL11 | 0.13 | 0.267 |
| Fas-L | 0.23 | 0.036* |
| IL-6 | 0.09 | 0.442 |
| IL-8 | 0.21 | 0.058 |
| IL-10 | 0.12 | 0.297 |
| IL-18 | 0.30 | 0.006* |
| TNFα | 0.01 | 0.937 |
| TNFSF14 | −0.04 | 0.722 |

CCL: CC chemokine ligand; CXCL: C-X-C motif chemokine;Fas-L: fas-ligand; IL: interleukin; TNF: tumor necrosis factor; TNFSF14: tumor necrosis factor superfamily member.*p < 0.05
