# Supplementary material for: CXCL9, CXCL10, and CXCL11; biomarkers of pulmonary inflammation associated with autoimmunity in patients with collagen vascular diseases–associated interstitial lung disease and interstitial pneumonia with autoimmune features
Source: PLoS One. 2020 Nov 2;15(11):e0241719. doi: 10.1371/journal.pone.0241719 (PMC7605704; doi:10.1371/journal.pone.0241719)
Supplement: S3 Table — CXCL: C-X-C motif chemokine; %FVC: percent predicted forced vital capacity; %DLco: percent predicted diffusing capacity of the lung for carbon monoxide; A–aDO2: alveolar-arterial oxygen difference; CRP: C-reactive protein; BALF: bronchoalveolar lavage fluid. *p < 0.05. (DOCX) [file pone.0241719.s003.docx]

S3 Table. Associations between baseline CXCL9, CXCL10, and CXCL11 levels and clinical characteristics in the CVD–ILD, IPAF, and IPF groups.

a. Serum CXCL9, CXCL10, and CXCL11 levels

|  | rs | | | | | | | | |
| --- | --- | --- | --- | --- | --- | --- | --- | --- | --- |
|  | %FVC | %DLco | A–aDO_2_ | CRP | BALF  Macrophages% | BALF  Lymphocytes% | BALF  Neutrophils% | BALF  Eosinophils% | BALF  CD4/8 ratio |
| CVD–ILD |  |  |  |  |  |  |  |  |  |
| CXCL9 | −0.14 | 0.05 | 0.15 | 0.25 | −0.13 | 0.02 | 0.26 | −0.10 | −0.27 |
| CXCL10 | −0.30 | 0.04 | 0.24 | 0.23 | −0.02 | −0.22 | 0.48 | −0.27 | −0.47 |
| CXCL11 | −0.46 | −0.19 | 0.23 | 0.08 | −0.30 | 0.20 | 0.14 | 0.10 | −0.25 |
| IPAF |  |  |  |  |  |  |  |  |  |
| CXCL9 | −0.18 | −0.16 | 0.22 | 0.45* | −0.44* | 0.36* | 0.26 | 0.19 | −0.42* |
| CXCL10 | −0.21 | −0.34 | 0.39* | 0.40* | −0.43* | 0.40* | 0.19 | 0.50* | −0.42* |
| CXCL11 | −0.31 | −0.20 | 0.41* | 0.31 | −0.45* | 0.39* | 0.40* | 0.17 | −0.32 |
| IPF |  |  |  |  |  |  |  |  |  |
| CXCL9 | −0.09 | 0.02 | 0.11 | 0.15 | −0.24 | 0.32* | 0.27 | −0.29 | 0.14 |
| CXCL10 | −0.22 | 0.03 | 0.24 | 0.14 | 0.00 | 0.11 | 0.21 | −0.23 | 0.26 |
| CXCL11 | −0.22 | 0.12 | 0.04 | 0.27 | −0.39* | 0.45* | 0.21 | −0.07 | 0.39* |

b. BALF CXCL9, CXCL10, and CXCL11 levels

|  | rs | | | | | | | | |
| --- | --- | --- | --- | --- | --- | --- | --- | --- | --- |
|  | %FVC | %DLco | A–aDO_2_ | CRP | BALF  Macrophages% | BALF  Lymphocytes% | BALF  Neutrophils% | BALF  Eosinophils% | BALF  CD4/8 ratio |
| CVD–ILD |  |  |  |  |  |  |  |  |  |
| CXCL9 | −0.14 | 0.38 | −0.54* | −0.25 | −0.16 | 0.40 | −0.35 | 0.48 | 0.39 |
| CXCL10 | 0.03 | −0.06 | −0.34 | −0.40 | −0.40 | 0.41 | −0.56* | 0.79* | 0.46 |
| CXCL11 | 0.01 | 0.24 | 0.11 | 0.09 | −0.01 | −0.15 | −0.35 | 0.24 | 0.11 |
| IPAF |  |  |  |  |  |  |  |  |  |
| CXCL9 | −0.16 | 0.01 | 0.38* | 0.27 | −0.90* | 0.64* | 0.69* | 0.52* | −0.05 |
| CXCL10 | −0.07 | 0.09 | 0.24 | 0.31 | −0.63* | 0.53* | 0.56* | 0.33 | 0.05 |
| CXCL11 | −0.07 | −0.14 | 0.22 | 0.01 | −0.11 | −0.15 | 0.35 | −0.03 | 0.20 |
| IPF |  |  |  |  |  |  |  |  |  |
| CXCL9 | −0.15 | 0.01 | 0.21 | 0.16 | −0.46* | 0.47* | 0.10 | 0.28 | 0.41* |
| CXCL10 | −0.09 | −0.02 | 0.21 | 0.15 | −0.37* | 0.41* | 0.05 | 0.16 | 0.41* |
| CXCL11 | 0.17 | 0.37* | 0.01 | −0.22 | −0.19 | 0.31 | 0.23 | −0.15 | 0.28 |

CXCL: C-X-C motif chemokine; %FVC: percent predicted forced vital capacity; %DLco: percent predicted diffusing capacity of the lung for carbon monoxide; A–aDO_2_: alveolar-arterial oxygen difference; CRP: C-reactive protein; BALF: bronchoalveolar lavage fluid. *p < 0.05.
