# Supplementary material for: CXCL9, CXCL10, and CXCL11; biomarkers of pulmonary inflammation associated with autoimmunity in patients with collagen vascular diseases–associated interstitial lung disease and interstitial pneumonia with autoimmune features
Source: PLoS One. 2020 Nov 2;15(11):e0241719. doi: 10.1371/journal.pone.0241719 (PMC7605704; doi:10.1371/journal.pone.0241719)
Supplement: S4 Table — CXCL: C-X-C motif chemokine;SP: surfactant protein; KL: krebs von den lungen. *p < 0.05. (DOCX) [file pone.0241719.s004.docx]

S4 Table. Associations between serum CXCL9, CXCL10, and CXCL11 levels and main IPF biomarkers levels.

|  | rs | | |
| --- | --- | --- | --- |
|  | SP-A | SP-D | KL-6 |
| CXCL9 | 0.01 | 0.12 | 0.12 |
| CXCL10 | 0.15 | 0.18 | 0.26* |
| CXCL11 | 0.15 | 0.20* | 0.17 |

CXCL: C-X-C motif chemokine;SP: surfactant protein; KL: krebs von den lungen. *p < 0.05.
