# Supplementary material for: CXCL9, CXCL10, and CXCL11; biomarkers of pulmonary inflammation associated with autoimmunity in patients with collagen vascular diseases–associated interstitial lung disease and interstitial pneumonia with autoimmune features
Source: PLoS One. 2020 Nov 2;15(11):e0241719. doi: 10.1371/journal.pone.0241719 (PMC7605704; doi:10.1371/journal.pone.0241719)
Supplement: S5 Table — CXCL: C-X-C motif chemokine. *p < 0.05. (DOCX) [file pone.0241719.s005.docx]

S5 Table. Associations between serum CXCL9, CXCL10, and CXCL11 levels and the severity of IPF

|  | %FVC | | p-value |
| --- | --- | --- | --- |
|  | 80%≤ | 80%< |  |
| CXCL9, pg/ml | 28.4 (17.1–47.9) | 30.9 (20.3–63.4) | 0.586 |
| CXCL10, pg/ml | 88.3 (46.7­–145.2) | 65.4 (20.4–164.5) | 0.500 |
| CXCL11, pg/ml | 0.0(0.0–70.5) | 0.0 (0.0–1832) | 0.338 |

CXCL: C-X-C motif chemokine. *p < 0.05.
