# Supplementary material for: CXCL9, CXCL10, and CXCL11; biomarkers of pulmonary inflammation associated with autoimmunity in patients with collagen vascular diseases–associated interstitial lung disease and interstitial pneumonia with autoimmune features
Source: PLoS One. 2020 Nov 2;15(11):e0241719. doi: 10.1371/journal.pone.0241719 (PMC7605704; doi:10.1371/journal.pone.0241719)
Supplement: Attachment — Submitted filename: PONE-Comments ver2.docx [file pone.0241719.s006.docx]

Reviewer #1: The authors evaluated the cytokine levels in serum and BALF, and found that serum CXCL9, CXCL10, and CXCL11 are useful biomarkers for autoimmune inflammation and predictors of immunosuppressant responses in ILD. Although the effect of the anti-fibrotic drug, nintedanib, on progressive fibrosing ILD has been already established, anti-inflammatory drugs are also effective in some ILD, including CVD-ILD and IPAF. However, there is currently no established biomarkers and treatments for them (anti-inflammatory drug or anti-fibrotic drug, or both?). This study provides an important contribution to the biomarker for patients who use anti-inflammatory drugs.
-> We thank the reviewer 1 for the positive overall evaluation of our study.

The authors described that CXCL9, CXCL10, and CXCL11 are important for the diagnosis of CVD-ILD and IPAF and anti-inflammatory therapy. However, it is unclear how to use these biomarkers in clinical practice. It would be informative to discuss the clinical application of these biomarkers.
-> We appreciate the precise comments. We added the sentence below at lane 361-363.

These serum biomarkers may enable clinicians to more easily evaluate when and to whom to administer anti-inflammatory drugs with simple blood tests in ILD management.

L141 cytokiness→cytokines

-> We corrected.

P11L182 Indicate the actual measurement value in the results (same below)

-> We added the measured value in the results. Please confirm the lanes 178-190, 210-213, 228-231, 246-247, 253-267, and 296.

Reviewer #2: 
In this manuscript, Kameda and colleagues assessed serum and BALF levels of various cytokines, including CXCL9, CXCL10, and CXCL11, in patients with CVD-ILD, IPAF and IPF. They also assessed clinical significance of these cytokine levels (CXCL9, CXCL10, and CXCL11) in serum and BALF, regarding association of clinical variables, diagnoses and response to immunosuppressive therapy. They found that serum and BALF levels of CXCL9, CXCL10, and CXCL11 were elevated in patients with CVD-ILD or IPAF compared with IPF. Levels of these chemokine were correlated with several clinical variables. In 10 patients with CVD-ILD or IPAF who treated with corticosteroid and/or immunosuppressive agents, serum CXCL9 and CXCL11 levels were positively associated with annual FVC changes. According to these findings, they concluded that CXCL9, CXCL10 and CXCL11 may be biomarkers to evaluate autoimmune inflammation and to predict response to immunosuppressive treatments. Their findings highlight interesting aspects of distinct pathophysiology among CVD-ILD, IPAF and IFP, however, there are several concerns in this study. As described in the limitation by the authors, the number of the patients, especially patients with CVD-ILD, were very small. Additionally, CVD-ILD contain variety of diseases, such as RA, Ssc, PM, MCTD and SS, which have different pathophysiology, respectively. Moreover, the number of patients who treated by immunosuppressive agent were only 10, which was too small to indicate that serum CXCL9 and CXCL11 are potential biomarker to predict response to immunosuppressive therapy.
-> We thank the reviewer 2 for the comments and suggestions in our study.

The authors showed the association between CXCL9, CXCL10 and CXCL11 levels and clinical variables, such as %FVC, %DLCO, A-aDO2, CRP and BALF findings in all the patient in Table 4 (page 15, 16). As the levels of these markers differed among CVD-ILD, IPAF and IPF, the correlations between these markers and clinical variables may be different among the three groups. The author should explain these points or assess the correlations in each group, respectively.
-> We appreciate the comments and suggestions about the correlations between the markers and clinical variables. We also expected the correlations between these chemokines and clinical variables may be different between the CVD–ILD, IPAF, and IPF groups. However, we could not adjust pulmonary function and retrieved rate of BALF between groups because of retrospective study. The normal BALF retrieved rate is 40-70%, but the proportion of patients with BALF retrieved rate of 40% or less in the CVD-ILD group was higher than in the other groups. This made it difficult to evaluate the correlations in the CVD-ILD group. We could not assess differences in the correlations caused by background autoimmunity in a subgroup analysis (S3 Table). We need a subgroup analysis in the next prospective study. We added retrieved rate of BALF in Table1 and the sentence below at lane 343-349.

As the levels of CXCL9, CXCL10, and CXCL11 differed between the CVD–ILD, IPAF, and IPF groups, we expected the correlations between these chemokines and characteristics may differ between groups. However, because of retrospective study, we were unable to adjust pulmonary function and retrieved rates of BALF between the groups and could not assess differences in the correlations caused by background autoimmunity in a subgroup analysis (S3 Table).

The authors showed the association between serum CXCL9, CXCL10 or CXCL11 levels and FVC change in 10 patients who treated with corticosteroid and/or immunosuppressive agents in CVD-ILD and IPAF groups in Figure 2 (page 17). This study include 16 patients with CDV-ILD and 35 patients with IPAF. In that case, how the 41 patients in CVD-ILD and IPAF groups were treated? The majority of the patients (41 patients) in CVD-ILD and IPAF groups did not receive corticosteroid and/or immunosuppressive agents? It is unclear how the authors chose the 10 patients for the correlation analyses between serum markers and response to treatment?

-> We appreciate the precise comments. In this retrospective observational analysis, the number of samples were relatively low, especially in the immunosuppressive treatment group. We need to evaluate the role as the predictive biomarkers in the next prospective study. We added the sentence below at lane 381-384.

Twenty-seven of the 41 patients in the CVD–ILD and IPAF groups were treated with immunosuppressive drug, but only 10 of them had pre-treatment serum samples and respiratory function test data one year after treatment due to the retrospective study nature.
