# Supplementary material for: CXCL9, CXCL10, and CXCL11; biomarkers of pulmonary inflammation associated with autoimmunity in patients with collagen vascular diseases–associated interstitial lung disease and interstitial pneumonia with autoimmune features
Source: PLoS One. 2020 Nov 2;15(11):e0241719. doi: 10.1371/journal.pone.0241719 (PMC7605704; doi:10.1371/journal.pone.0241719)
Supplement: Attachment — Submitted filename: PONE-Comments ver3.docx [file pone.0241719.s007.docx]

Reviewer #1: I think the points that I pointed out last time have been improved.
One last comment, do you think that in the future you will not have to measure BALF if you measure CXCL9, CXCL10, and CXCL11 only with serum? From the results of this manuscript, I understood that. Add a vision of what to do when using this marker clinically in the future. I advise you to make the conclusion easier to understood.

-> We appreciate the additional comments. We tried to present the usefulness of these serum biomarkers for clinicians. We added the sentence below at lane 361-367 (discussion), 48-50(abstract conclusions) and 392-394 (conclusions).

In the future, monitoring of these serum biomarkers from the time of diagnosis could be useful in determining whether immunosuppressive therapy is initiated without BAL. In particular, when ILD worsens during follow-up, these biomarkers may enable clinicians to choose anti-inflammatory drugs or anti-fibrotic drugs.

Abstract conclusions

Serum CXCL9, CXCL10, and CXCL11 are potential biomarkers for autoimmune inflammation and predictors of the immunosuppressive therapy responses in ILD with background autoimmunity.

Conclusions

Serum CXCL9, CXCL10, and CXCL11 may reflect autoimmune inflammation of ILD and work as biomarkers to predict the response to immunosuppressive therapy in the management of ILD with background autoimmunity.

Reviewer #2: The authors have responded to the reviewer’s questions adequately. The manuscript has been improved.
Hope to validate their findings in future prospective studies.

-> We thank the reviewer 2 for the comments in our study. We plan to validate in the next prospective study.
